# A Rare Case of Irinotecan-induced Interstitial Pulmonary Disease and Diffuse Alveolar Hemorrhage in a Patient with Pancreatic Cancer

**DOI:** 10.7759/cureus.5041

**Published:** 2019-06-29

**Authors:** Lingbin Meng, Bo Deng, Baoqiong Liu, Umair Majeed, Wen Wang

**Affiliations:** 1 Internal Medicine, AdventHealth Winter Park, Winter Park, USA; 2 Internal Medicine, AdventHealth Orlando, Orlando, USA

**Keywords:** irinotecan, chemotherapy, interstitial lung disease, diffuse alveolar hemorrhage

## Abstract

Irinotecan is a cytotoxic medication used to treat various cancers. Diffuse alveolar hemorrhage (DAH) and interstitial pulmonary disease (IPD) are both serious and life-threatening complications, which are rarely reported to be associated with irinotecan. In our case, however, a pancreatic cancer patient developed both DAH and IPD after administration of irinotecan with fluorouracil (5FU).

## Introduction

Irinotecan is a cytotoxic medication classified as a plant alkaloid and topoisomerase I inhibitor. It has been approved for treating various cancers including colorectal, gastric, lung, and brain cancers. Diffuse alveolar hemorrhage (DAH) is a serious and life-threatening condition, which can be caused by systemic autoimmune diseases, toxins, transplantation, and medications. Common culprits include antiplatelet and anticoagulant medications. DAH is rarely associated with chemotherapeutic agents. To the best of our knowledge, only one case of irinotecan-induced DAH has been documented in the literature [1]. Although interstitial lung disease is often related to the use of chemotherapeutic medications. IPD is rarely a side effect of Irinotecan [2-4]. We herein report an interesting case of the development of both DAH and IPD in a pancreatic cancer patient after the administration of irinotecan with fluorouracil (5FU). 

## Case presentation

A 60-year-old male with a history of pancreatic cancer presented to our emergency department with a syncopal episode. He was diagnosed with stage IV pancreatic cancer and was started on chemotherapy with a combination of 5-FU and irinotecan (FLOFRI regimen) three months ago. His last chemotherapy was one week prior to admission. On admission, he was noted to have an oxygen saturation of 75% on 5L oxygen through a nasal cannula. He was then placed on heated high flow oxygen without improvement. The patient was subsequently intubated for worsening acute hypoxic respiratory failure.

**Figure 1:**
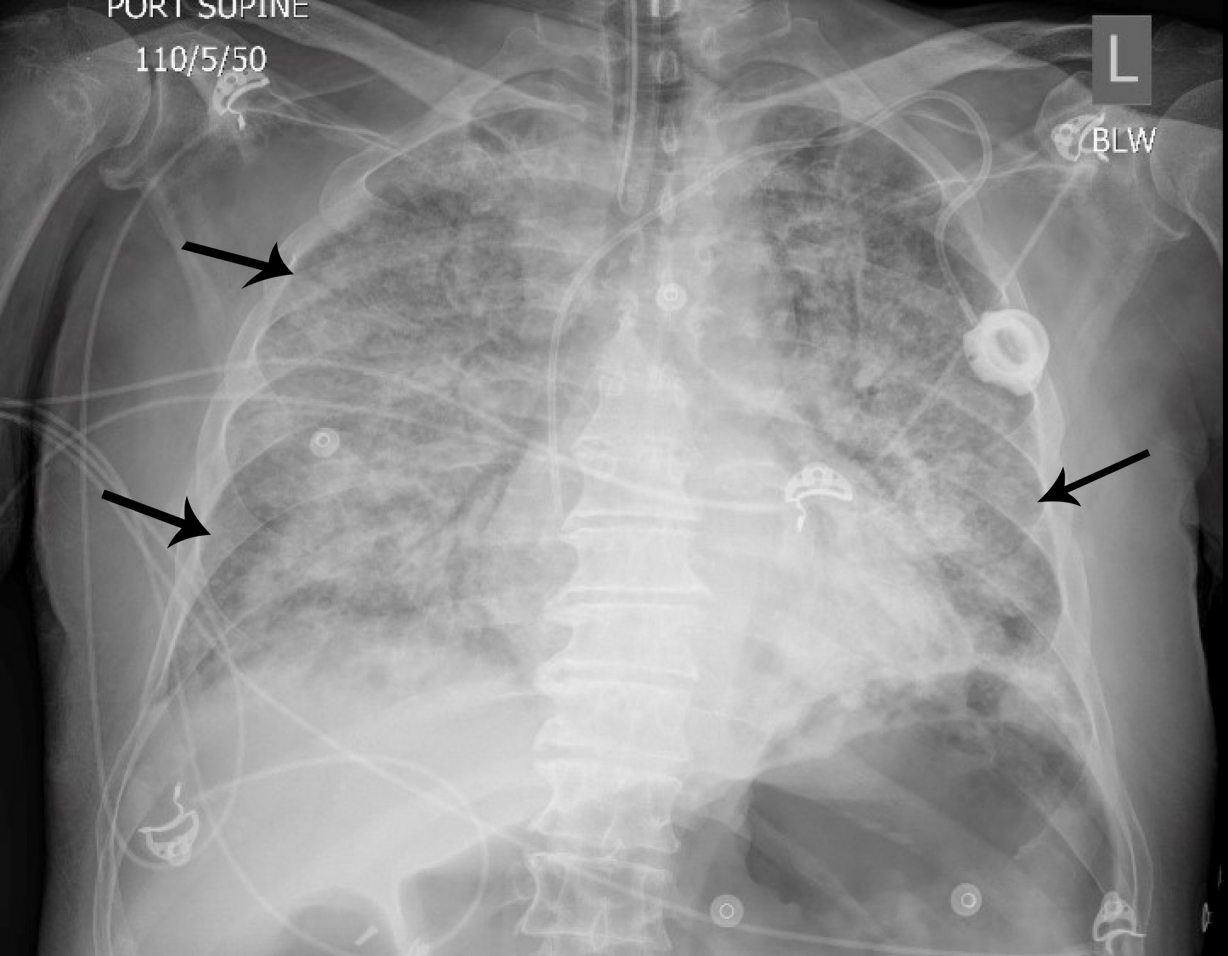
CXR shows diffuse bilateral lung infiltrates (black arrows) CXR, chest X-ray

**Figure 2:**
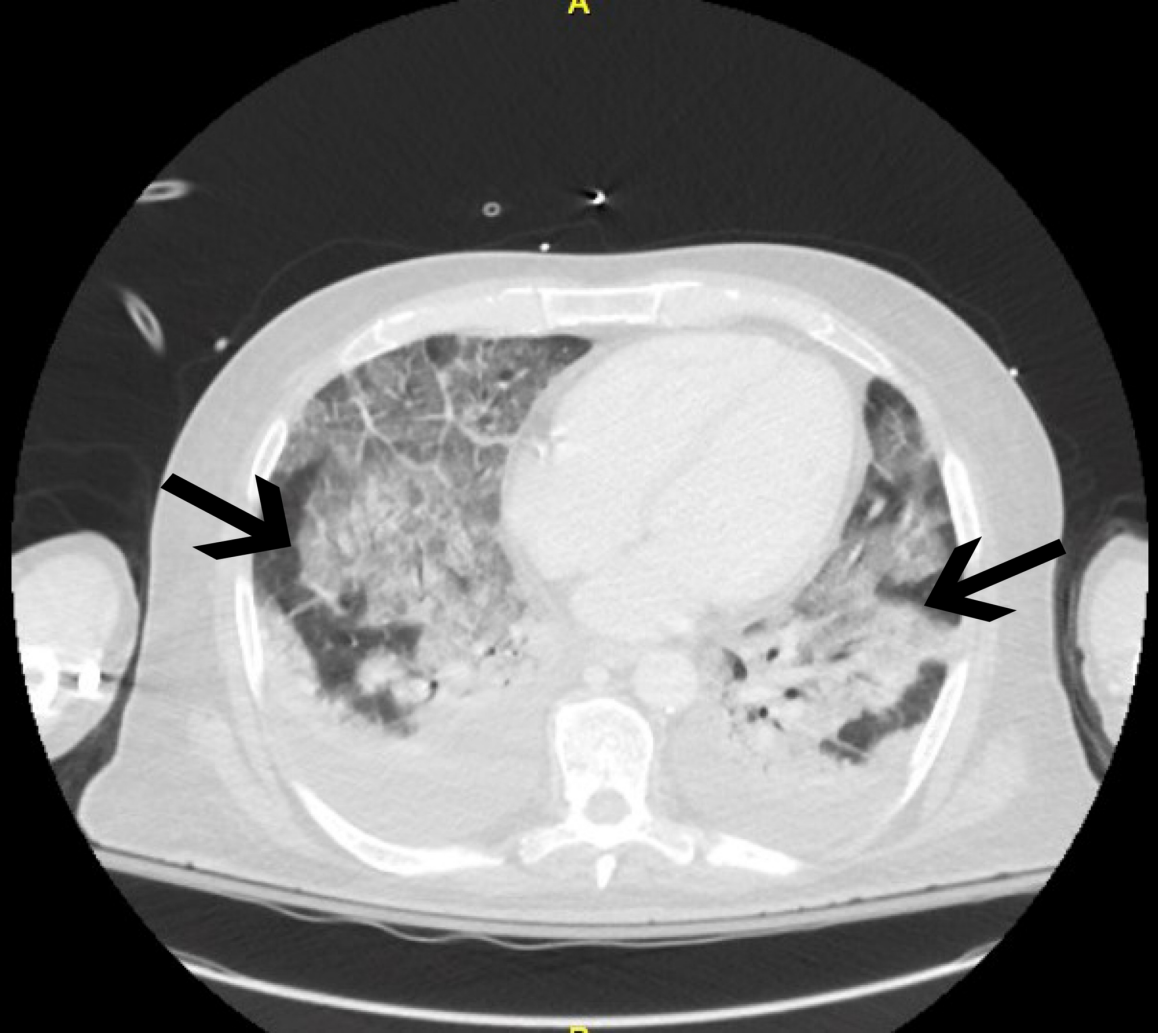
CT of chest with contrast demonstrates ground glass infiltrate and increased reticular markings (black solid arrows) in the dependent portion of both lungs suspicious for interstitial lung disease and possible early fibrosis

**Figure 3:**
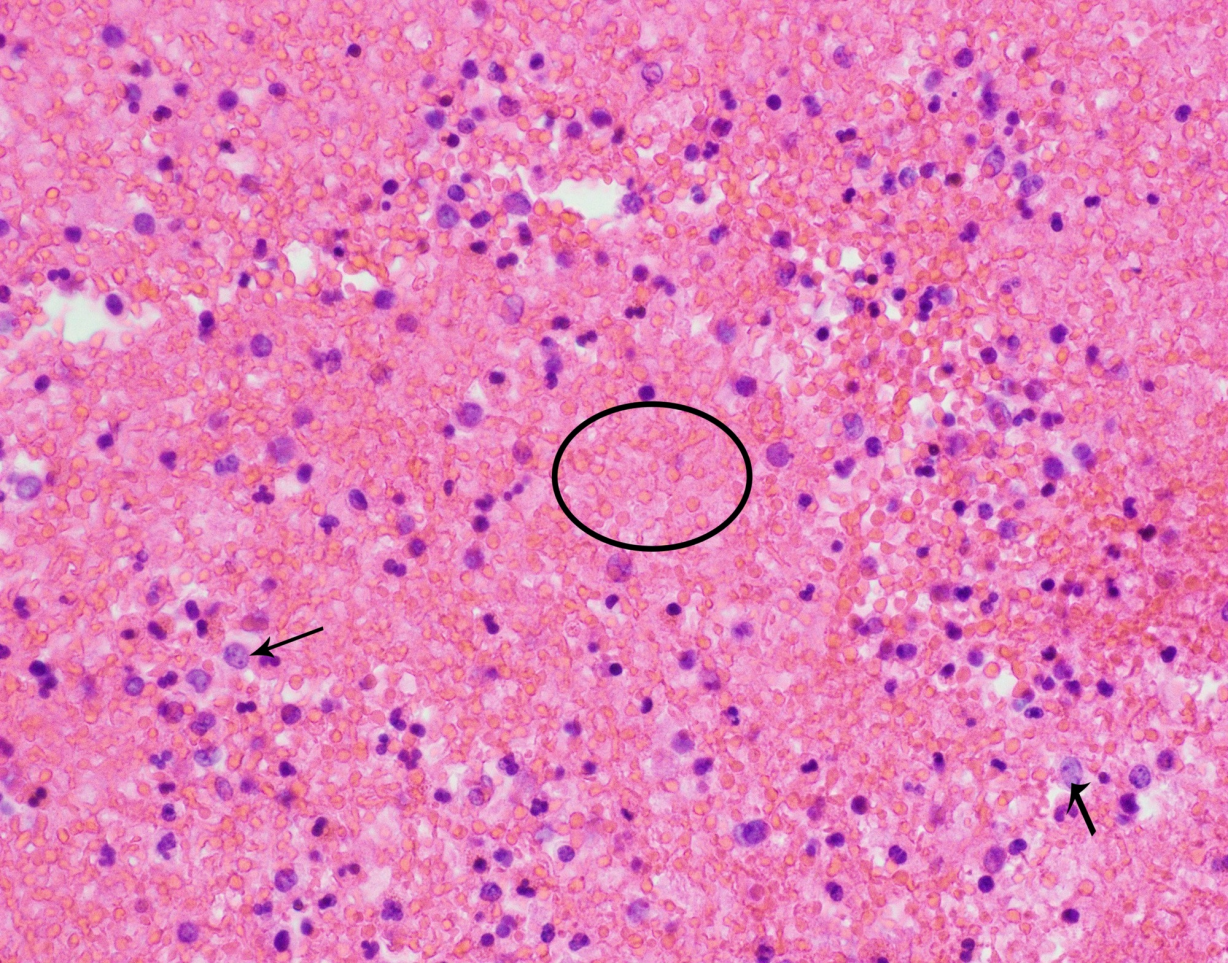
Bronchoscopy lavage smear shows scattered macrophages (black arrows) in the background of numerous red blood cells (marked with circle)

The physical examination showed diffused coarse breath sounds in bilateral lung fields. The complete blood count (CBC) revealed that the white blood cell count was 12,000 cells/L, with 12% of band cells, 81% of neutrophils, 1% of lymphocytes, 1% of monocytes, and 1% of eosinophils. A chest X-ray (CXR) showed diffuse bilateral infiltrates (Figure 1). Computed tomography (CT) of chest revealed subtle ground glass infiltrates and reticular markings suspicious for interstitial lung disease, along with two parenchymal nodules in the left lung (Figure 2). Blood cultures were negative. The urinary antigen of *Legionella* was negative. Bronchoscopy showed diffuse alveolar hemorrhage. Microscopic examination of the alveolar lavage sample showed large amounts of macrophages and red blood cells (Figure 3), confirming the diagnosis of DAH. Serology was negative for human immunodeficiency virus (HIV), viral hepatitis panel, anti-streptolysin O antibody (ASO), and C-reactive protein (CRP). Autoimmune panel was negative for antinuclear antibodies (ANA), anti-double-stranded DNA (anti-dsDNA), anti-citrullinated protein antibody (anti-CCP), perinuclear anti-neutrophil cytoplasmic antibodies(P-ANCA), anti-neutrophil cytoplasmic antibody (C-ANCA), anti-cardiolipin, anti-glomerular basement membrane (anti-GBM), and anti-topoisomerase antibody (anti-SCL70). Direct examination and culture of bronchoalveolar lavage fluid were negative for viruses, bacteria, fungi, mycobacteria, and *Pneumocystis jirovecii*. Serum polymerase chain reaction (PCR) also was negative for Aspergillus, *Cytomegalovirus* (CMV), *P. jirovecii*, and fungi. Cardiac ultrasound showed the ejection fraction of 60% to 65% with normal left ventricular diastolic function and mildly elevated right ventricular systolic pressure (RVSP) of 41 mmHg.

We held chemotherapy in light of the patient’s deteriorating condition. We started him on stress dose of steroid and broad-spectrum antibiotics, including cefepime, vancomycin, and azithromycin. The patient required prolonged ventilator support and had to receive tracheostomy as a result. His condition gradually improved after three weeks of supportive treatment. He was eventually extubated successfully. The family decided for hospice care, and the patient was transferred to the inpatient hospice service. 

## Discussion

This patient presented with sudden onset of interstitial pneumonia with diffuse alveolar hemorrhage, which was likely caused by his recent administration of the chemotherapeutic agents FLOFRI due to the time coincidence and exclusion of alternative explanations. The patient did not present with any symptoms or signs of connective tissue diseases or systemic autoimmune diseases. Laboratory data was negative for the series of auto-immunological markers. Infection workup was also negative. We did not perform biopsy because the patient was clinically ill and had a significant bleeding risk with a platelet count of 50,000 cells/L. Also, the cytology analysis of bronchial lavage revealed no evidence of cancer metastasis in the lung (Figure 3). Besides chemotherapy with 5FU and Irinotecan, the patient was not on any other medications that may have caused pulmonary toxicities. Overall, the recent chemotherapy with FLOFRI seemed to be directly associated with the development of IPD and DAH in this patient.

Irinotecan was shown to cause non-specific pulmonary toxicities in previous phase II clinical trials [5]. Interstitial lung disease is a well-known side effect of various chemotherapeutic medications. But both IDP and DAH are rare complications of Irinotecan administration. Multiple recent cases of irinotecan-induced interstitial pneumonia in treating colorectal cancers were reported in the literature, demonstrating IPD as one of the potential side effects of Irinotecan [2-4]. Only one previous case of irinotecan-induced DAH has been published, suggesting DAH is an extremely rare side effect of Irinotecan [1]. 5FU could also be the potential cause; however, 5FU has almost never been accompanied by pulmonary toxicities. There has been only one report of pulmonary side effects associated with administration of 5FU [6]. Therefore, we consider irinotecan to be the cause of IPD and DAH in this patient, although the contribution of 5FU cannot be completely excluded. 

The underlying mechanism of irinotecan-associated IPD and DAH is largely unknown, although direct cytotoxic effect and inflammatory response, such as hypersensitivity-mediated reaction, may play a role. It was postulated that irinotecan, as an isotopomerase inhibitor, can directly inhibit DNA synthesis or repair, inducing cytotoxic effects in pneumocytes and/or the interstitial cells. The cytology of bronchial lavage in this patient revealed the infiltration of large amounts of macrophages, suggesting the involvement of an inflammatory response. Our patient and previously published irinotecan-induced IPD or DAH patients all required systemic steroid therapy, indicating that longer duration of systemic steroids as an adjuvant therapy with Irinotecan or slower tapering of systemic steroids may reduce the incidence of irinotecan-induced IPD or DAH. Further studies are required to test this possibility and identify the exact underlying mechanisms of irinotecan-induced IPD or DAH.

## Conclusions

To the best of our knowledge, our study is the first case report showing irinotecan could possibly induce both IPD and DAH in a pancreatic cancer patient. DAH is a life-threatening situation that requires immediate discontinuation of Irinotecan therapy and administration of systemic steroids. Longer duration of systemic steroids or slower tapering of systemic steroids may reduce the incidence of serious pulmonary adverse events of irinotecan. It is effective for both IPD and DAH. Further studies are required to clarify whether it is a direct toxic effect or a hypersensitivity-mediated reaction.
